# Experiment and Modelling of Ultrasonic Vibration-Assisted Creep-Aging Tensile for 7055-T6 Alloy

**DOI:** 10.3390/ma19112223

**Published:** 2026-05-25

**Authors:** Duquan Zuo, Haoran Fu, Tianyu Xu, Ti Ye, Yanjie Han, Chong Gao

**Affiliations:** 1 College of Aviation Engineering, Civil Aviation Flight University of China, 46, Nanchang Road, Guanghan 618307, China; 2 School of Engineering, University of Tokyo, 7-3-1 Hongo, Bunkyo-ku, Tokyo 113-8656, Japan

**Keywords:** ultrasonic vibration, creep-aging tensile, volume effect, mechanical properties

## Abstract

This study investigates the effects of ultrasonic vibration on the creep-aging tensile behavior of 7055-T6 aluminum alloy through experiments and finite element simulations. Two characteristic parameters—effective softening amplitude (ESA) and recovery amplitude (RA)—are introduced to quantify the competing softening and hardening effects induced by ultrasonic vibration. Experimental results reveal that the maximum ESA (28.1 MPa) occurs at an amplitude of 14.01 μm, whereas optimal plasticity is achieved at 12.53 μm, indicating that maximum softening does not coincide with optimal formability. Intermittent vibration enhances creep plastic strain by up to 6.95% at 12.53 μm, contrasting with the detrimental effect of continuous vibration. A viscoplastic constitutive model incorporating the volumetric effect of ultrasonic vibration is developed and validated via finite element simulations, achieving close agreement with experiments (ESA deviation ≤ 1.9 MPa). These findings provide quantitative guidance for parameter optimization in ultrasonic-assisted creep-aging formation.

## 1. Introduction

The 7055 aluminum alloy is a critical material for lightweight structural applications in the aerospace industry [1,2,3]. To fully exploit its advantages and broaden its application scope, simultaneous improvement in both strength and plasticity is required to meet the demands of modern large aircraft fuselage panel forming and service performance [4]. However, an inherent trade-off exists between strength and plasticity in metallic materials [5]. To overcome this limitation and enhance forming precision, considerable attention has been devoted to investigating the creep characteristics of aluminum alloys. The creep-aging tensile (CAT) test serves as a key component in the constitutive modeling of creep-aging forming for large-scale fuselage panels [6]. Liu et al. [7] found in their study on the effects of T6 treatment on the properties of the 7055 alloy and other 7xxx series aluminum alloys that elevated temperatures induce the transformation of η′ phase into η phase within the 7xxx aluminum alloys, leading to a decrease in metal strength. Therefore, post-process optimization is crucial for unlocking the full mechanical potential of aluminum alloys. Hong et al. [8] conducted CAT and room-temperature tensile experiments on 7075-T6 thin plates and reported a significant reduction in residual stress after creep-aging forming. Zuo et al. [9] introduced an electrostatic field into the aging forming process of 7050 alloy and observed that electrostatic field-assisted solution treatment reduced the springback rate while increasing the elongation of the aged components.

In parallel, the incorporation of ultrasonic vibration into creep-aging tensile testing—referred to as ultrasonic vibration-assisted creep-aging tensile (UVCAT) testing—has been shown to reduce internal stresses during forming and improve the overall mechanical properties of the formed material. For instance, Duan et al. [10] investigated the effect of ultrasonic vibration on 7075 aluminum plates after creep-aging forming and found that it significantly reduced the springback rate and enhanced the mechanical properties.

In the context of ultrasonic-assisted forming, Shirmendagva et al. [11] compared conventional progressive forming with its ultrasonic-assisted counterpart and reported that the introduction of ultrasound reduced the springback effect and the forming force. Liu et al. [12] investigated the effects of ultrasonic vibration on the wire drawing of titanium and elucidated its role in plastic deformation. They found that the stress superposition effect induced by ultrasonic vibration generates an oscillatory stress field within the material, which partially counteracts the internal stresses and thereby reduces the residual stress in the formed component. Ma et al. [13] examined the influence of ultrasonic vibration on the tensile properties of titanium foils and observed a significant decrease in flow stress accompanied by an increase in elongation during ultrasonic loading. In addition, they reported a non-monotonic variation in flow stress, which initially decreased and then increased over the course of ultrasonic vibration.

Considerable research has focused on the effect of ultrasonic vibration on the deformation behavior of aged components [8,14,15,16]; however, the mechanisms by which ultrasonic vibration influences the forming force during creep-aging remain poorly understood [17]. Furthermore, the role of ultrasonic vibration parameters in modulating the softening effect is still unclear, which hinders stable deformation control in the forming process. Accordingly, further investigation is required into the influence of vibration parameters and modes on the mechanical properties and deformation behavior during the creep-aging tensile (CAT) process.

To address this gap, the present study investigates the effects of ultrasonic vibration parameters and modes on the CAT process using 7055-T6 aluminum alloy. By integrating ultrasonic vibration with creep-aging tensile testing, ultrasonic vibrations with varying amplitudes are applied during the deformation of the alloy. The study compares specimens subjected to continuous vibration, intermittent vibration, and no vibration, and evaluates the influence of ultrasonic vibration on forming force, tensile strength, creep elongation, and microstructure. The findings are expected to provide a reference for the application of ultrasonic vibration-assisted creep-aging tensile (UVCAT) processes.

## 2. Materials and Experiments

**Figure 1:**
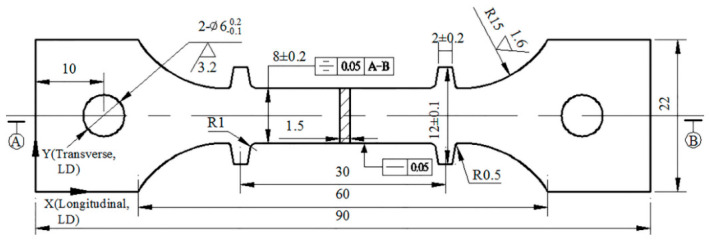
Specimen for creep-aging tensile test. (Unit: mm) [20].

The hot-rolled 7055 aluminum alloy used in this study has the chemical composition listed in Table 1. Tensile specimens were prepared in accordance with the ISO 204:2023 standard [18,19]. The specimen geometry is illustrated in Figure 1, and the material properties are summarized in Table 2.

**Figure 2:**
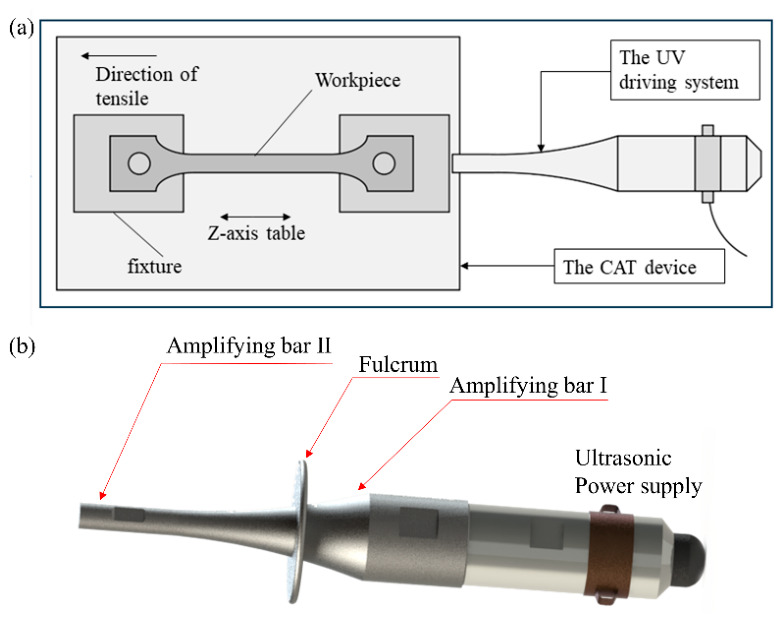
Experimental device schematic diagram and physical diagram: (**a**) ultrasonic-assisted creep-aging tensile test schematic diagram; (**b**) Vibratory sub-physical diagram.

**Figure 3:**
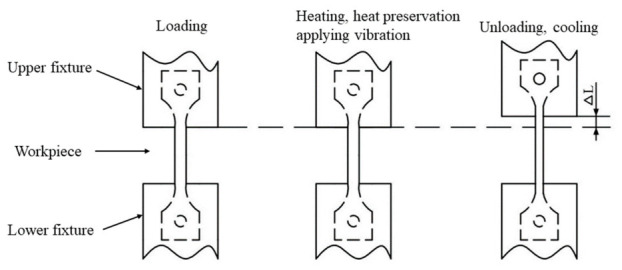
The schematic diagram of the UVCAT principle.

During testing, the specimen was fixed using a combination of pins and shoulder clamps. The upper end of the clamp was connected to a loading lever, while the lower end was screwed to the vibrator rod, as schematically shown in Figure 2. The working principle of the ultrasonic vibration-assisted creep-aging tensile (UVCAT) setup is illustrated in Figure 3.

The temperature during the tests was controlled by a Pinsheng electric thermostatic blast drying oven (Vertical model 101-1s, manufactured by Pinsheng, Shaoxing, Zhejiang, China). Ultrasonic vibration was introduced into the specimen through a Huiyi Ultrasonic 20K2600W device (Huiyi Ultrasonic, Changzhou, Jiangsu, China), which served as the core of the vibration system. The ultrasonic generator operates at a frequency of 20 kHz with a rated power of 2600 W. In this study, four ultrasonic power levels were employed: 1000, 1500, 2000, and 2500 W, corresponding to amplitudes of 8.86, 10.85, 12.53, and 14.01 μm, respectively, based on previously established calibration data [21,22,23].

To investigate the effect of ultrasonic vibration on the creep-aging behavior, two experimental groups were designed. Group 1 was subjected to conventional creep-aging tensile (CAT) testing, while Group 2 underwent ultrasonic vibration-assisted CAT (UVCAT) testing. The UVCAT tests were further categorized into continuous and intermittent vibration modes under the same frequency but varying amplitudes.

**Figure 4:**
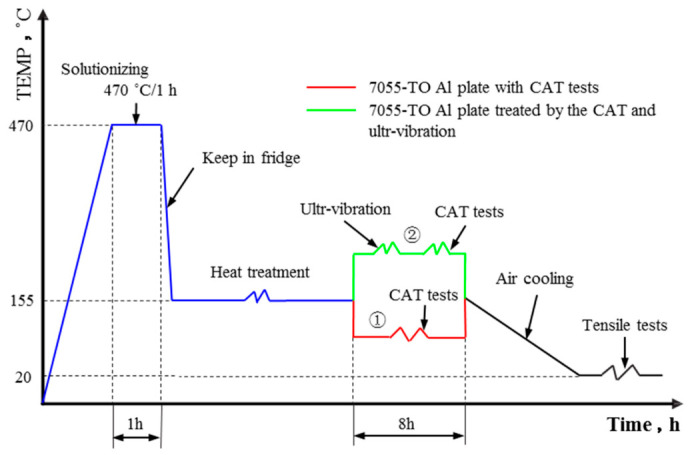
Schematic diagram of creep-aging tensile test scheme.

As illustrated in Figure 4, Group 1 comprised two T6-tempered specimens tested without ultrasonic vibration (indicated by the red line in Figure 4). Group 2 consisted of 16 T6-tempered specimens subjected to UVCAT testing (indicated by the green line in Figure 4). The vibration schemes are summarized in Table 3, and each condition was tested in duplicate to ensure reproducibility.

All 18 specimens were tested under identical creep-aging conditions, with a tensile stress of 130 MPa, a temperature of 155 °C, and a holding time of 8 h. After the creep-aging treatment, the specimens were subjected to room-temperature tensile testing at a constant crosshead speed of 2 mm/min. Subsequently, specimens for scanning electron microscopy (SEM) observation were sectioned from the fractured samples to examine the fracture morphology.

## 3. Results and Analysis

### 3.1. Materials Forming Force Under Ultrasonic Vibration

**Figure 5:**
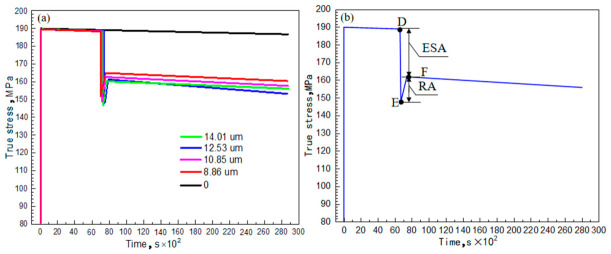
Stress–time curve and forming force–time calibration diagram of non-vibration and continuous vibration with different amplitudes. (**a**) Real stress–time curve. (**b**) ESA, RA calibration.

Figure 5a shows the variation of true stress with time for the alloy under different ultrasonic amplitudes during the process of deformation elongation from 0% to a maximum of 7%. Upon the application of ultrasonic vibration combined with creep-aging, the flow stress exhibits an instantaneous linear decrease, indicating a pronounced softening effect in the 7055 aluminum alloy. Following this initial drop, the flow stress reaches a minimum value and then undergoes a brief increase before gradually stabilizing. During the stable stage, the flow stress curves show a slow decline, with those obtained under vibration remaining nearly parallel to the curve without vibration, albeit at considerably lower stress levels.

As also shown in Figure 5a, with increasing vibration amplitude, the creep time corresponding to the instantaneous stress drop initially increases and then decreases. Meanwhile, the onset of the stable flow stress stage shifts to shorter creep times at higher amplitudes. Notably, at an amplitude of 14.01 μm, the instantaneous stress drop reaches a maximum value of 160.3 MPa at a creep time of 7210 s, at which the softening effect is most pronounced.

Based on the true stress–time curve shown in Figure 5b, two characteristic parameters are defined. The Effective Softening Amplitude (ESA) is defined as the difference between the stress at the onset of vibration (Point D) and the stress at the onset of steady-state flow stress (Point F). The Recovery Amplitude (RA) is defined as the difference between the stress at the minimum of the abrupt linear drop that follows Point D (Point E) and the stress at Point F. The ESA and RA values were calculated for each vibration amplitude.

As shown in Figure 5b, upon the application of ultrasonic vibration, the flow stress decreases instantaneously and linearly to Point E, followed by a brief recovery and increase to Point F, after which the stable UVCAT stage begins. This trend is consistently observed across all tested amplitudes.

### 3.2. Material Strength Under Ultrasonic Vibration

**Figure 6:**
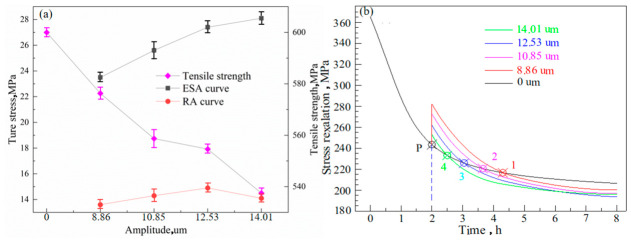
The creep curves and tensile strength of ESA and RA with amplitude and stress relaxation with time. (**a**) Creep change and tensile strength change curve; (**b**) Stress relaxation curve.

Table 4 summarizes the ESA, RA, and tensile strength as a function of vibration amplitude. The corresponding curves are plotted in Figure 6a. As shown in Figure 6a, the ESA increases with amplitude following an initially rapid increase that gradually decelerates. At an amplitude of 8.86 μm, the ESA reaches a minimum value of 23.5 MPa, corresponding to a relative softening of 12.47%. At an amplitude of 14.01 μm, the ESA reaches a maximum of 28.1 MPa, corresponding to a relative softening of 14.91%. When the amplitude exceeds 12.53 μm, the ESA decreases and approaches a plateau, indicating that the material’s susceptibility to softening exhibits amplitude-dependent behavior.

This trend can be attributed to the competing effects of ultrasonic vibration on the viscoplastic deformation behavior. At lower amplitudes, the applied ultrasonic energy effectively promotes dislocation mobility and enhances the softening effect, leading to a reduction in flow stress. However, when the amplitude becomes excessively high, the increased energy dissipation associated with viscoplastic deformation dampens the efficiency of stress superposition, thereby attenuating the softening effect of ultrasonic vibration on the creep-aging tensile process.

As shown in Figure 6a, the RA exhibits relatively little variation with increasing amplitude. This is likely attributed to the transient relative displacement between the fixture and the specimen upon the application of ultrasonic vibration, which causes the flow stress to drop to its minimum value. Following this, the fixture–specimen system stabilizes, and after a slight stress recovery, the UVCAT process enters its stable stage. The small variation in RA across different amplitudes suggests that the post-vibration stabilization behavior is largely independent of amplitude within the tested range.

As also shown in Figure 6a, the tensile strength decreases monotonically with increasing vibration amplitude. Specifically, in the amplitude range of 0–10.85 μm, the reduction in tensile strength is relatively pronounced. Between 10.85 and 12.53 μm, the decrease becomes less pronounced, whereas beyond 12.53 μm, the rate of reduction increases again. This nonlinear trend may be explained by the competing effects of ultrasonic vibration on the material’s deformation behavior. High-amplitude ultrasonic vibration introduces increased energy dissipation during viscoplastic deformation, which can impede dislocation motion and thus reduce the material’s plastic deformation capability, ultimately leading to a decrease in tensile strength.

Figure 6b presents the stress relaxation curves, representing the evolution of residual stress over time during the creep-aging tensile process, for specimens tested with and without ultrasonic vibration. As shown in Figure 6b, for the specimen without vibration, the residual stress decreases rapidly in the initial stage and then more gradually with increasing creep time. This behavior is attributed to the influence of the creep strain rate on internal stress relaxation.

Ultrasonic vibration was applied when the creep strain reached 6.5‰ (Point P in Figure 6b). Regardless of the applied amplitude, the residual stress curves for the vibrated specimens exhibit a similar trend—an initial rapid decrease followed by a slower decline—and consistently lie below that of the specimen without vibration. This indicates that the applied ultrasonic vibration accelerates stress relaxation by rapidly reducing internal stresses arising from deformation incompatibility within the microstructure.

Upon the application of vibration, an instantaneous increase in the stress relaxation curve is observed. The rate of this instantaneous increase decreases with increasing amplitude, suggesting that higher-amplitude vibration introduces more energy, facilitating faster elimination and homogenization of internal stresses during the initial stage.

As summarized in Figure 6b, the final residual stress values at the endpoint are 205.8, 200.2, 196.3, 193.8, and 196.1 MPa for amplitudes of 0, 8.86, 10.85, 12.53, and 14.01 μm, respectively. Overall, the application of ultrasonic vibration reduces the residual stress. Within the amplitude range of 8.86–12.53 μm, a larger amplitude leads to more complete residual stress release. However, when the amplitude exceeds 12.53 μm, the residual stress exhibits a slight increase, a trend consistent with the nonlinear behavior observed for tensile strength.

### 3.3. Deformation Characteristics of Materials

In CAT tests, the creep plastic strain (*ε*_p_) serves as a key indicator for evaluating the viscoplastic behavior of the material. It is defined as the sum of the initial plastic strain (*ε_i_*) and the subsequent creep strain (*ε_f_*), where *ε_f_* represents the percentage increase in the gauge length at time t under the specified temperature relative to the original gauge length [24].

In the present study, the viscoplastic deformation is enhanced by the synergistic effect of ultrasonic vibration and creep. Specifically, creep promotes stress relaxation, while the stress superposition effect induced by ultrasonic vibration softens the material and facilitates plastic flow. This synergistic interaction promotes the conversion of elastic deformation into inelastic deformation, thereby leading to more substantial release of internal residual stresses.

**Figure 7:**
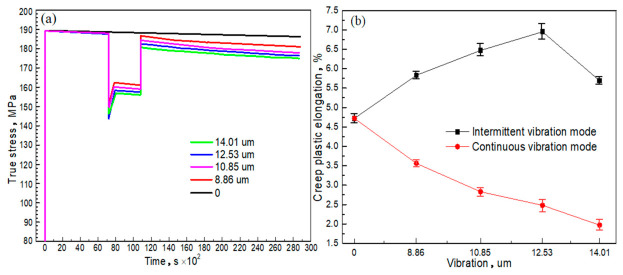
Stress and creep plastic elongation curves under intermittent vibration. (**a**) Real stress–time curve; (**b**) Creep plastic elongation curves under different amplitudes.

As observed in the continuous vibration experiments, a hardening-like effect manifests at larger amplitudes, characterized by an increase in flow stress and residual stress (Figure 6a,b). To investigate the influence of this effect on material plasticity, intermittent UVCAT experiments were conducted. In these tests, ultrasonic vibration was applied for 1 h upon reaching the secondary creep stage—specifically, after 2 h of creep time—and was then immediately ceased. The resulting true stress–time curve is presented in Figure 7a.

In this experiment, the creep plastic strain (*ε*_p_) was measured using a gauge length marking method [25] and was calculated for both continuous and intermittent vibration conditions. The results are summarized in Table 5, and the corresponding *ε*_p_ versus amplitude curves are presented in Figure 7b.

As shown in Figure 7b and Table 5, under continuous vibration, *ε*_p_ decreases markedly with increasing amplitude. This is attributed to the pronounced hardening-like effect induced by prolonged ultrasonic vibration, which becomes more significant at larger amplitudes, thereby reducing the creep plastic strain. In other words, sustained ultrasonic vibration impairs the viscoplastic behavior of the creep-aging tensile specimens.

In contrast, intermittent vibration exhibits a different trend. As the amplitude increases from 8.86 to 12.53 μm, *ε*_p_ increases. However, when the amplitude exceeds 12.53 μm, *ε*_p_ decreases sharply. For instance, at an amplitude of 14.01 μm, *ε*_p_ is 0.14% lower than that at 8.86 μm. This non-monotonic behavior suggests that moderate vibration amplitudes promote viscoplastic deformation under intermittent conditions, whereas excessive amplitudes may introduce hardening effects that counteract the softening enhancement.

**Figure 8:**
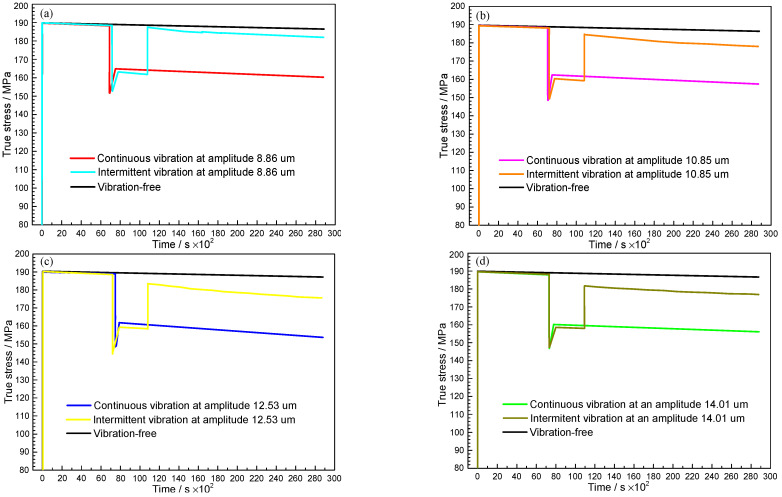
Real creep stress–time curves under different loading modes. (**a**) 8.86 μm; (**b**) 10.85 μm; (**c**) 12.53 μm; (**d**) 14.01 μm.

Figure 8 presents the true stress–time curves for continuous and intermittent vibration modes. Compared with the curves for the non-vibrated and continuously vibrated specimens (Figure 7a), the flow stress curve under intermittent vibration lies at a lower level. Furthermore, under intermittent vibration, the endpoint flow stress decreases with increasing amplitude. These observations indicate that reducing the ultrasonic excitation time delays the onset of the hardening-like phenomenon typically associated with prolonged continuous vibration.

The above analysis indicates that excessive vibration amplitude or prolonged ultrasonic vibration duration reduces the creep plastic strain (*ε*_p_) of the creep-aging tensile specimens. Conversely, the appropriate selection of vibration amplitude and duration enhances the viscoplastic deformation capability of the material.

### 3.4. Microstructure Plastic Deformation

**Figure 9:**
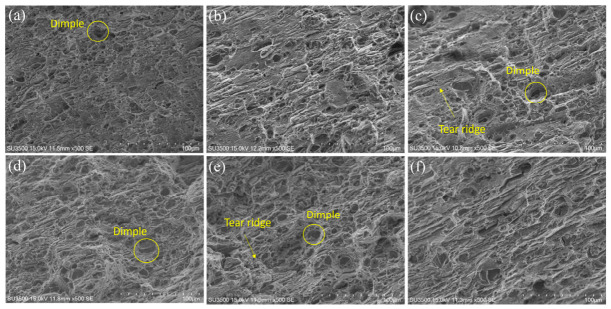
Fracture morphology of specimens without UVCAT and UVCAT-treated specimens; (**a**) UVCAT-untreated 7055-T6; (**b**) 0 μm; (**c**) 8.86 μm; (**d**) 10.85 μm; (**e**) 12.53 μm; (**f**) 14.01 μm.

As shown in Figure 9a, the fracture surface of the pre-stretched 7055-T6 alloy without UVCAT treatment exhibits numerous equiaxed dimples of varying sizes. Figure 9b–f present scanning electron microscopy (SEM) images of the fracture surfaces of age-hardened tensile specimens tested under different amplitudes at 500× magnification.

As observed in Figure 9c–f, the fracture surfaces exhibit dimples of varying sizes, along with tear ridges, second-phase particles, and shear steps of varying heights, indicating a mixed-mode fracture characteristic. For the specimen tested at an amplitude of 8.86 μm (Figure 9c), the fracture morphology is dominated by elongated dimples that are more numerous and deeper than those in the non-vibrated specimen (Figure 9b), with a few second-phase particles present, although some dimples remain relatively large.

At an amplitude of 10.85 μm (Figure 9d), the dimples become more uniform and equiaxed. They are more numerous and smaller in size, with tear ridges present at the dimple bottoms. Second-phase particles are more frequently distributed around the dimples, while the dimple depth is shallower compared with that at 8.86 μm.

With a further increase in amplitude to 12.53 μm (Figure 9e), the fracture morphology reveals numerous oval dimples accompanied by a few shear steps. Tear ridges are visible within the dimples, and second-phase particles are more densely distributed around them. The dimples are significantly more numerous, deeper, smaller, and more uniformly distributed than those observed at lower amplitudes, suggesting grain refinement under the action of ultrasonic vibration. This refined microstructure is consistent with the enhanced viscoplastic behavior and the most pronounced softening effect observed at this amplitude, as indicated by the mechanical testing results (Figure 5a and Figure 6a).

## 4. Constitutive Model and Simulation

### 4.1. Constitutive Model

UVCAT is a hybrid forming process that integrates ultrasonic vibration into CAT. The forming behavior becomes inherently complex due to the coupling of multiple mechanisms, including stress relaxation, creep, dynamic stress effects, and springback [26,27]. To achieve precise control over this hybrid forming process, it is necessary to account not only for the elastic and plastic deformation of the alloy but also for the viscous deformation arising from these mechanisms. By incorporating elastic components into the Bingham model [28,29], a simplified elastoviscoplastic constitutive model can be expressed as follows:

(1) $$\left\{\begin{array}{c} \dot{\varepsilon }=E^{-1}\dot{\sigma }+\eta^{-1}\left(\sigma -\sigma_{p}\right),\sigma >\sigma_{p}\\ \dot{\varepsilon }=E^{-1}\dot{\sigma },\sigma \leq \sigma_{p} \end{array}\right.$$

Based on the elastoviscoplastic physical model, the viscoelastic Kelvin model is incorporated into the viscoplastic Bingham model, resulting in the following viscoplastic constitutive model:

(2) $$\left\{\begin{array}{c} \varepsilon \left(t\right)=E^{-1}\left[1-H\left(t\right)\right],\sigma >\sigma_{s}+K\varepsilon_{vp}\\ \varepsilon \left(t\right)=\varepsilon_{0}+E^{-1}\sigma_{0}\left[1-H\left(t\right)\right]+C_{1}H\left(t\right)+{\eta_{1}}^{-1}\eta_{2}\left[\sigma \left(t\right)-\sigma_{s}\right],\sigma \leq \sigma_{s}+K\varepsilon_{vp} \end{array}\right.$$

In the equation, *H*(t) = exp(−η_1_^−1^*Et*). Considering the combined mechanisms of subsequent yielding, strain, strain rate, and elastic strain, the dynamic stress function based on viscoelastic plasticity can be derived as:

(3) $$\left\{\begin{array}{c} \sigma \left(t\right)={(\eta }_{1}\eta_{2}/\eta_{1}{+\eta }_{2})[\dot{\varepsilon }\left(t\right)+E\varepsilon_{e}\left(t\right)/\eta_{1}+\sigma_{s}+K\varepsilon_{vp}(t)/\eta_{2}]\\ \varepsilon_{e}\left(t\right)=E^{-1}\sigma_{0}\left[1-H\left(t\right)\right]\\ \varepsilon \left(t\right)=\varepsilon_{0}t+Asin2\pi ft\\ \varepsilon_{vp}=\varepsilon \left(t\right)-\varepsilon_{0}-\varepsilon_{e} \end{array}\right.$$

In the equation, *ε_e_*(*t*) is the integral of Equation (2), and *ε_vp_*(*t*) is the viscoplastic function, with *η*_1_ and *η*_2_ representing the viscosity strain coefficients of the elastic and plastic components, respectively. The above viscoplastic constitutive model reveals the mechanism of the volume effect in ultrasonic vibration-assisted creep-aging forming of aluminum. From the equation, it can be seen that the vibration frequency is directly proportional to the dynamic stress. When dynamic stress is excited by low-frequency ultrasonic vibration, it leads to an average stress that is lower than the deformation stress under quasi-static conditions. Additionally, the stress superposition effect caused by ultrasonic vibration is more significant at low frequencies.

In the UVCAT forming process, no surface effects exist. Therefore, based on the volume effect, the viscoplastic rheological model of 7055 aluminum under cyclic stress in one cycle, derived from dislocation theory, is as follows:

(4) $$\left\{\begin{array}{c} \varepsilon =\varepsilon_{0}t+Asin2\pi ft\\ d\sigma_{pk}/dN=4\varepsilon_{a}\kappa \left(1-\sigma_{pk}/\sigma_{pks}\right)t\\ \kappa =0.5\alpha Mk_{1}Gb\sigma_{ss}/\sigma_{ss}-\sigma_{0},\sigma_{ss}={C_{ss}(1-\bar{I_{pk}})}^{m_{3}}\\ \dot{\sigma }\;\left(\varepsilon \right)=E,\sigma [\dot{\sigma }\;\left(\varepsilon \right)]<0\\ \dot{\sigma }\;\left(\varepsilon \right)=\kappa_{d}\left[1-\lambda \sigma /\sigma_{ss}\right],\sigma \left[\dot{\sigma }\left(\varepsilon \right)\right]>0,\left|\sigma \right|>0 \end{array}\right.$$

In the equation, when *σ* > 0, *κ_d_* = −*κ*, when *σ* < 0, *κ_d_* = *κ*, *ε*_0_ quasi-static strain, A and *σ_a_* are the amplitude and excitation stress, respectively.

### 4.2. Boundary Conditions and Fe Modelling

In the finite element modeling, the tensile specimen is divided into three sections: left, middle, and right. The left and right sections of the gauge length are set as analytical rigid bodies, while the middle section is set as a deformable body. The true stress–strain and creep strain data of the material can be obtained through experiments, and then, by using Equation (5), the required plasticity parameters for the simulation can be calculated. The formula for plastic strain *ε_pl_* is as follows:

(5) $$\varepsilon_{pl}=\varepsilon^{\prime }-\varepsilon_{cr}-\varepsilon_{e}=\varepsilon^{\prime }-\varepsilon_{cr}-\sigma /E$$

In the equation, *σ* and *ε′* represent the true stress and true strain, respectively, *ε_e_* is the elastic strain, E is the elastic modulus, and *σ_cr_* is the creep strain. The ultrasonic vibration signal is applied using the *Amplitude/Periodic type, with periodic motion applied to the right end of the specimen according to the amplitude curve (Equation (6)) [30].

(6) $$a=A_{0}+\sum_{n=1}^{N}A_{n}\cos nw\left(t-t_{0}\right)+B_{n}\sin nw\left(t-t_{0}\right)$$

Given the minimal influence of interfacial friction during the UVCAT process, the effect of vibration on contact friction is neglected, i.e., surface effects are considered negligible. In the UVCAT simulation, vibration frequency and amplitude are treated as two independent variables to analyze their influence on the creep strain and stress during the creep tensile process, thereby enabling investigation of the stress superposition effect associated with the volumetric effects of ultrasonic vibration.

The simulation parameters are set as follows: creep tensile rate of 1 mm/min, creep temperature of 155 °C, and creep stress of 130 MPa. Ultrasonic vibration is applied after 2 h of creep, maintained continuously for 1 h, and then immediately ceased. The total creep tensile duration is 8 h. Based on the experimental conditions described above, the designed UVCAT simulation scheme is summarized in Table 6. The simulation software and version used is Abaqus 2020.

### 4.3. Results and Analysis

**Figure 10:**
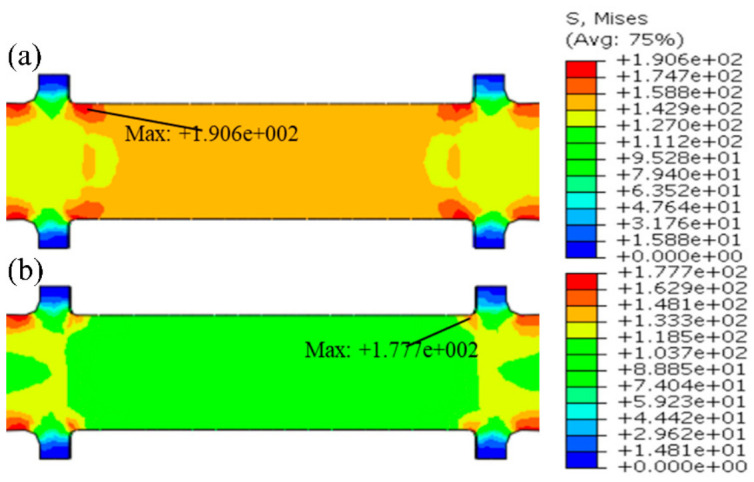
Stress distribution nephogram of amplitude specimen. (**a**) 0 M; (**b**) 12.53 M.

Figure 10 presents the stress distribution contours for the non-vibrated specimen and the specimen tested with a vibration amplitude of 12.53 μm. By comparing the simulation results with the experimental values, it can be observed that before unloading, the maximum stress in the non-vibrated specimen is 190.6 MPa, which is 4.4 MPa higher than the experimental value of 186.2 MPa. For the vibrated specimen, the simulated maximum stress is 177.7 MPa, which is 1.5 MPa higher than the experimental value of 176.2 MPa. The stress distribution also reveals a significant reduction in material stress under ultrasonic vibration, which is attributed to the softening effect induced by the vibration.

The comparison indicates that the discrepancies between the simulation results and the experimental data are within the acceptable engineering range, demonstrating that the established finite element model accurately describes the UVCAT process for the 7055 aluminum alloy.

**Figure 11:**
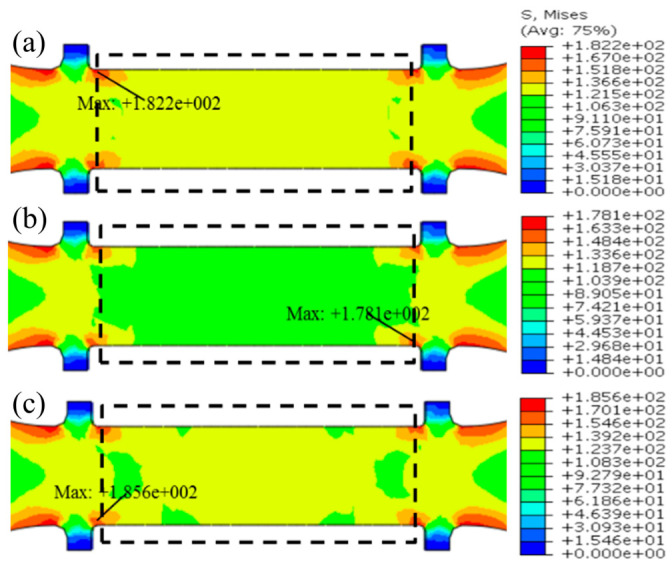
Stress distribution cloud diagram under different amplitudes. (**a**) 8 μm; (**b**) 12 μm; (**c**) 16 μm.

Figure 11 presents the stress distribution contours of the tensile specimens under different amplitude conditions. Focusing on the specimen gauge section (outlined by the black dashed line) in conjunction with Figure 10a, the overall stress distribution appears broadly similar across the different amplitudes. However, the material’s susceptibility to softening exhibits amplitude-dependent behavior. The maximum stress values for amplitudes of 0, 8, 12, and 16 μm are 190.6, 182.2, 178.1, and 185.6 MPa, respectively. Evidently, the maximum stress decreases initially and then increases with increasing amplitude, reaching a minimum at an amplitude of 12 μm.

**Figure 12:**
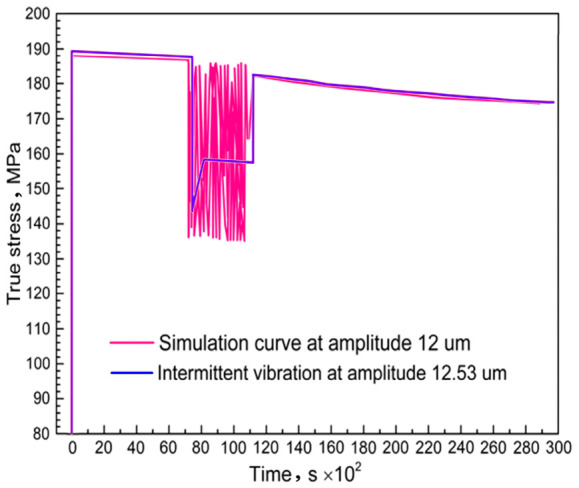
12.53 μm and 12 μm stress–time curve.

Figure 12 presents the true stress–time curves for the 7055 alloy during the UVCAT process, obtained from both experimental measurements and finite element simulations under comparable amplitude conditions. Both approaches employed a vibration frequency of 20 kHz, with an amplitude of 12.53 μm in the experiment and 12 μm in the simulation. The experimental data reflect the evolution of average stress over time under ultrasonic vibration, while the simulation data at an amplitude of 12 μm represent the stress superposition effect induced by the vibration.

Comparing the two curves, upon the application of ultrasonic vibration, the flow stress exhibits an instantaneous and significant decrease. When the vibration ceases, the average flow stress shows an instantaneous recovery, after which the stress curve returns to a level approximately consistent with that of the creep tensile test without vibration. In the experiment, the ESA, defined as the reduction in average stress induced by vibration, is 28.4 MPa. In the simulation, the oscillation amplitude of the stress following vibration is 53.0 MPa, with an average stress of 161.5 MPa, yielding an ESA of 26.5 MPa. The difference between the simulated and experimental ESA values is 1.9 MPa.

**Figure 13:**
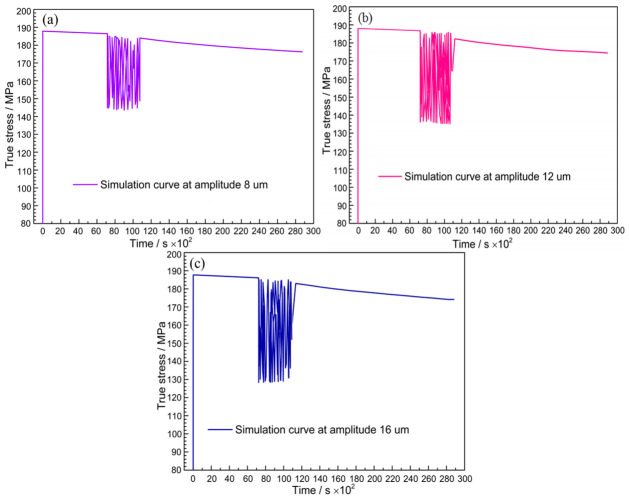
The real stress–time diagram in the simulation process of creep tensile parts under different amplitudes at 20 kHz. (**a**) 8 μm; (**b**) 12 μm; (**c**) 16 μm.

Figure 13 presents the simulated true stress–time curves for the creep tensile specimens under a vibration frequency of 20 kHz and varying amplitudes. Regardless of the applied amplitude, upon the superposition of ultrasonic vibration, the stress exhibits an instantaneous drop followed by a periodic oscillation process. When the vibration ceases, the stress rises instantaneously and quickly recovers to the level observed in the creep tensile specimen without vibration.

As the amplitude increases, the stress oscillation amplitude becomes more pronounced. For vibration amplitudes of 8, 12, and 16 μm, the oscillation amplitudes are 41.6, 53.0, and 55.6 MPa, with corresponding average stress values of 164.6, 161.5, and 156.7 MPa, respectively. The corresponding effective softening magnitudes (ESM) are 20.8, 26.5, and 27.8 MPa, respectively. For comparison, the experimentally determined ESM values at vibration amplitudes of 8.86 and 14.01 μm are 26.4 and 31.2 MPa, respectively, showing reasonable agreement with the simulated trends.

A comparison of the results reveals a notable discrepancy in the ESA between the experimental and simulation results. At a small amplitude (8 μm in the simulation vs. 8.86 μm in the experiment), the simulated ESA is considerably higher than the experimental value. In contrast, at larger amplitudes (>12 μm), the two sets of results are in reasonable agreement.

This discrepancy can be attributed to the fact that the finite element simulation captures only the stress superposition effect, whereas the reduction in flow stress observed experimentally arises from multiple contributing factors, including acoustic softening and thermally induced softening, in addition to stress superposition. Consequently, at small amplitudes, these additional factors exert a significant influence on the alloy’s flow behavior. Under moderate amplitude conditions, the stress superposition effect becomes the dominant mechanism. However, when the amplitude is excessively large, the effectiveness of stress superposition diminishes, leading to a less pronounced softening effect.

**Figure 14:**
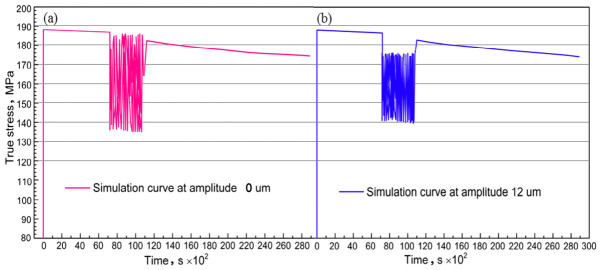
The stress–time diagram of the material constitutive model under different experimental modes at 20 kHz and 12 μm. (**a**) Non-vibration CAT experimental; (**b**) UVCAT experimental.

To elucidate the vibration-induced softening phenomenon in the simulation, the stress–strain data obtained from the ultrasonic vibration-assisted CAT experiment were employed as the constitutive model for the material, using a vibration frequency of 20 kHz and an amplitude of 12 μm as an example, while keeping other parameters unchanged. Figure 14a presents the stress–time curves obtained by applying different frequencies and amplitudes after establishing the material’s constitutive model based on the stress–strain data measured from the CAT experiment. Figure 14b shows the stress–time relationship obtained through finite element simulation using the constitutive model constructed under the UVCAT experimental mode.

As shown in Figure 14b, when the constitutive model established under the UVCAT experimental mode is applied in the simulation, the stress oscillation amplitude is 36.4 MPa, the average stress is 158.2 MPa, and the corresponding ESA is 27.9 MPa. In contrast, using the constitutive model derived from the non-vibrating CAT experiment yields an ESA of 26.5 MPa. From the previous UVCAT experimental results, under vibration parameters of 20 kHz and 12.53 μm, the experimentally determined ESA is 28.4 MPa.

A comparison of the results indicates that the simulation using the constitutive model derived from UVCAT experimental data produces results that are in closer agreement with the experimental values than those obtained using the CAT-based constitutive model. This suggests that employing stress–strain data from UVCAT experiments as the material’s constitutive model enables a more accurate representation of the hybrid aging forming behavior in simulations.

## 5. Discussion

**Figure 15:**
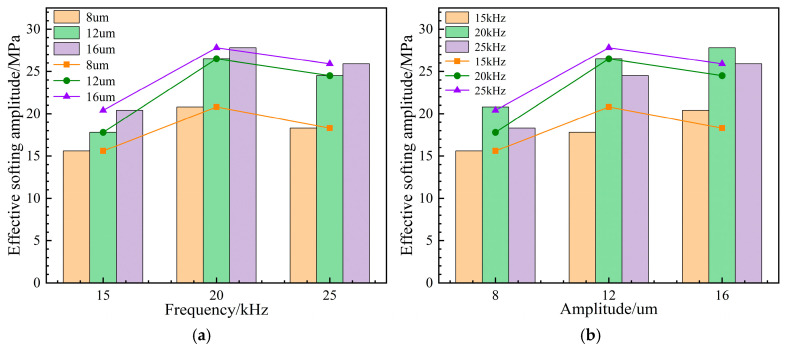
ESA changes with frequency and amplitude. (**a**) Under different amplitudes; (**b**) Under different frequencies.

Table 7 summarizes the simulated ESA of the material’s flow stress under different frequencies and amplitudes, and the corresponding ESA variation curves are presented in Figure 15. As shown in Table 7 and Figure 15, at a given amplitude, the ESA initially increases and then decreases with increasing vibration frequency. Additionally, regardless of the frequency, the ESA exhibits a consistent increasing trend with amplitude. These findings are in good agreement with the experimental results, with the maximum ESA occurring at a vibration frequency of 20 kHz.

In the UVCAT experiments, by controlling two sets of variables—amplitude (vibration frequency) and excitation mode—it is found that ultrasonic vibration exerts two distinct effects on the plastic forming capability of creep specimens, with the dominance of softening and hardening effects varying with amplitude.

From the perspective of forming force, the softening effect is characterized by an instantaneous linear decrease in flow stress upon the application of ultrasonic vibration, followed by a brief recovery before entering the stable UVCAT stage. The ESA increases rapidly at first and then more gradually, a trend that remains consistent regardless of the excitation mode. When the vibration ceases, the flow stress recovers to the level of the non-vibrated specimen during tensile testing, indicating that no permanent effect is induced by ultrasonic vibration.

From the perspective of creep plastic strain, the hardening effect becomes evident under prolonged excitation or high-amplitude vibration. In such cases, the flow stress decreases, the residual stress increases, and the creep plastic strain (*ε*_p_) decreases, indicating reduced material plasticity. Conversely, under short-duration or low-amplitude vibration, εp increases to varying extents, reflecting improved plasticity. This behavior aligns with the residual effect documented by Langenecker [31]: when ultrasonic vibrations are stopped, no aftereffect is observed at low intensity, whereas when the intensity exceeds a critical value that depends on the chosen material, “residual hardening” occurs upon termination of the vibrations.

From the perspective of fracture morphology, the 7055 aluminum alloy without UVCAT treatment exhibits numerous equiaxed dimples of uneven sizes. In tensile tests conducted with and without ultrasonic vibration, the grains are elongated or fractured, and the fracture surfaces display elongated honeycomb-like structures with a few fractured second-phase particles at the dimple bottoms, characteristic of ductile fracture. As the amplitude increases from 0 to 14.01 μm, the extent of ductile fracture initially increases and then decreases, with the most pronounced ductile fracture observed at an amplitude of 12.53 μm, where the material exhibits the best plasticity. In other words, with increasing amplitude, the material’s plasticity first increases and then decreases, which is in good agreement with the conclusions drawn from the true stress–time curves obtained during creep tensile testing.

## 6. Conclusions

By conducting UVCAT experiments on 7055-T6 aluminum alloy, the effects of ultrasonic vibration on the forming force, tensile strength, creep plastic strain, and microstructure of creep tensile specimens were analyzed. The main conclusions are as follows:

1. Quantification of softening-hardening competition. Ultrasonic vibration induces an instantaneous linear decrease in flow stress upon application, followed by a brief recovery and entry into the stable UVCAT stage. The ESA increases with amplitude up to 14.01 μm (maximum of 28.1 MPa, relative softening of 14.91%), beyond which the softening effect diminishes. Notably, the amplitude corresponding to maximum ESA (14.01 μm) differs from that yielding optimal plasticity (12.53 μm), indicating that maximum softening does not equate to optimal formability.
2. Excitation mode governs viscoplastic response. Under continuous vibration, creep plastic strain (*ε*_p_) decreases monotonically with increasing amplitude, reflecting a dominant hardening effect. In contrast, intermittent vibration promotes *ε*_p_, which increases from 5.83% at 8.86 μm to 6.95% at 12.53 μm, before decreasing at higher amplitudes. This demonstrates that short-duration vibration enhances plasticity, whereas prolonged excitation impairs it.
3. Fracture morphology correlates with mechanical behavior. Ductile fracture, characterized by equiaxed and oval dimples with tear ridges and second-phase particles, is most pronounced at 12.53 μm, consistent with the optimal plasticity observed in mechanical testing. This amplitude corresponds to the finest and most uniformly distributed dimple structures, suggesting grain refinement under ultrasonic vibration.
4. Validation of constitutive model and simulation. A viscoplastic constitutive model incorporating the volumetric effect of ultrasonic vibration was established and validated via finite element simulations. The model reproduces the experimental stress–time response with high accuracy (ESA deviation within 1.9 MPa). Combining simulation and experimental results, the optimal parameters for industrial UVCAT are: amplitude 12.53 µm, intermittent mode, and frequency 20 kHz.

## Figures and Tables

**Table 1:** Chemical composition (mass fraction, %) of 7055 aluminum alloy [20].

Zn	Mg	Cu	Zr	Fe	Mn	Si	Ti	Cr	Al
7.9	2.1	2.3	0.05	0.15	0.05	0.1	0.06	0.001	Bal.

**Table 2:** Mechanical properties of 7055-T6 aluminum alloy at room temperature.

Density (g/cm^3^)	μ	E/GPa	Rm/MPa	Yield Strength/MPa
2.7	0.3	69	509	411

**Table 3:** UVCAT experimental scheme of 7055-T6 aluminum plate.

Number	Temperature (°C)	Time (h)	Amplitude (μm)	Drawing Speed	Excitation Mode
1	155	8	0	1 mm/min	-
2	155	8	8.86	1 mm/min	When the creep is 6.5‰, the vibration is excited until the end of stretching
3	155	8	10.85	1 mm/min	
4	155	8	12.53	1 mm/min	
5	155	8	14.01	1 mm/min	
6	155	8	8.86	1 mm/min	Excitation when the aging time is 2 to 3 h
7	155	8	10.85	1 mm/min	
8	155	8	12.53	1 mm/min	
9	155	8	14.01	1 mm/min	

**Table 4:** The variation of ESA, RA, and tensile strength with amplitude.

Amplitude (μm)	ESA (MPa)	RA (MPa)	Tensile Strength (MPa)
0	-	-	600.0
8.86	23.5	13.6	576.3
10.85	25.6	14.3	558.7
12.53	27.4	14.9	554.6
14.01	28.1	14.1	537.5

**Table 5:** Creep plastic elongation at different amplitudes under different loading modes.

Amplitude (μm)	0	8.86	10.85	12.53	14.01
Continuous vibration mode (%)	4.72	3.56	2.83	2.48	1.97
Intermittent vibration mode (%)	4.72	5.83	6.47	6.95	5.69

**Table 6:** Simulation scheme for UVCAT.

Number	Frequency (kHz)	Amplitude (μm)	Number	Frequency (kHz)	Amplitude (μm)
1	-	-	6	20	12
2	15	8	7	20	16
3	15	12	8	25	8
4	15	16	9	25	12
5	20	8	10	25	16

**Table 7:** Simulation of effective softening amplitude of flow stress at different frequencies and amplitudes (unit: MPa).

Frequency (kHz)	Amplitude (μm)		
	8	12	16
15	15.6	17.8	20.4
20	20.8	26.5	27.8
25	18.3	24.5	25.9

## Data Availability

The original contributions presented in this study are included in the article. Further inquiries can be directed to the corresponding author.
